# An Account of the Practice of One of the Physicians of the Westminster General Dispensary, and of the Western Dispensary, from the 20th of June, to the 20th of July

**Published:** 1806-08

**Authors:** S. Fothergill

**Affiliations:** Southampton Street, Strand


### *An Account of the Practice of one of the Physicians of the Westminster General Dispensary, and of the Western Dispensary, from the 20th of June, to the 20th of July.*

| | | | |
|---|---|---|---|
| ACUTE DISEASES. | | Cholera - - - | 3 |
| Synochus - - - | 5 | Intestinal Hæmorrhage | 1 |
| Catarrh - - - | 1 | Acute Diseases of Infants | 8 |
| Peripneumony - - | 3 | CHRONIC DISEASES. | |
| Pleurisy - - - | 2 | Pulmonary Consumption | 4 |
| Acute Rheumatism - | 3 | Marasmus - - | 2 |
| Hæmoptoe - - | 1 | Cough and Dyspnœa | 17 |
| Sore-Throat - - | 2 | Asthma - - - | 1 |
| Erysipelas - - | 2 | Pleurodyne - - | 2 |

Asthenia

| | | | |
|---|---|---|---|
| Asthenia - - | 9 | Enlargement of the Heart | 1 |
| Hypochondriasis - | 2 | Menorrhœa - - | 2 |
| Chronic Rheumatism | 7 | Chlorosis - - | 1 |
| Lumbago and Sciatica | 3 | Amenorrhœa - - | 4 |
| Cephalalgia - - | 2 | Leucorrhœa - - | 2 |
| Hemiplegia - - | 1 | Hysteria - - - | 3 |
| Hemicranium - - | 2 | Prolapsus Uteri - | 1 |
| Epilepsy - - - | 3 | Scirrhus of the Uterus | 1 |
| Ascites and Anasarca | 3 | Lues - - - | 4 |
| Dyspepsia - - | 2 | Lepra Venerea - | 2 |
| Enterodynia - - | 3 | Psora - - - | 1 |
| Diarrhœa - - - | 8 | Porrigo - - - | 1 |
| Worms - - - | 2 | Herpes - - - | 1 |
| Hæmorrhoids - - | 1 | | |

The acute diseases which have been under my care during the last month were not severe; those which have not already terminated favourably are going on well.

A very frequent complaint in the summer season is synochus. This fever continues from ten days to three and four weeks, is not contagious, and often arises from using too great exertion during the heat of the day. It is preceded by languor, heaviness, and debility; a general sense of chill succeeded by increased heat of the skin and a quick pulse; pain in the head and occasionally delirium; general restlessness, anxiety, and depression of mind; with a frequent, and at times painful respiration; a white, and in severe cases, brown and parched tongue; great thirst and aversion to food; are the symptoms which usually denote the presence and progress of this disease. The state of the bowels is various; in some cases diarrhœa continues during most of the time, while in others costiveness requires attention; the urine is generally of a high colour. As far as my experience goes, young men are the most frequent subjects of this fever; though neither age nor sex is exempt. In the cure of this mild species of fever, one great object is not to do harm, by administering violent medicines, or reducing the vital powers by bleeding and copious evacuations. To keep up a gentle moisture on the skin, to encourage the patient to drink freely of cool diluting liquids, and to avoid costiveness, will generally be found sufficient; where however the debility is great, and the delirium urgent, more powerful remedies, as the preparations of bark, camphor, or opium, may be exhibited. The cold affusion would most probably shorten the period of the fever, and often entirely stop its progress; but poor people when attended at their own habitations, in general, dislike dislike the practice, and neglect the proper application of a remedy which has our warmest admiration; and whose discoverer merits the general gratitude of mankind.

Paralysis, which most frequently attacks people who have passed the middle period of life, and those chiefly who have been addicted to intemperance, sometimes visits the young and innocent.

The case of hemiplegia reported this month, occurred in a girl five years old. She was seized in the night with a fit, and continued two hours in a state of insensibility; when a little recovered she was unable to articulate, and had lost the use of the arm and leg of the right side. A week afterwards she had a second fit, which continued six hours. Previous to her first attack she complained of pain and heaviness in her head, and three days after the last fit was delirious for several hours. About this time she became a patient at the Dispensary; her arm and leg hung useless by her side, and she could not speak; these were the only perceptible indications of disease, and I could not discover any probable cause for the fits. Knowing that the bowels of children are frequently disordered, and that from their want of proper action, complaints apparently unconnected with them are sometimes produced, I gave every other morning twelve grains of pulvis scammonii cum calomelane, and in ten days the child was completely well; a blister to the nape of the neck, and friction, were the only other applications used during the time of her being at the Dispensary.

*Southampton Street, Strand, July 22, 1806.* S. FOTHERGILL.

